# Aqueous Bark Extract of *Ceiba speciosa* (A. St.-Hill) Ravenna Protects against Glucose Toxicity in *Caenorhabditis elegans*

**DOI:** 10.1155/2020/1321354

**Published:** 2020-10-08

**Authors:** Fabrine Bianchin dos Santos, Caroline Brandão Quines, Luiz Eduardo Ben Pilissão, Ana Helena de Castro Dal Forno, Cristiane Freitas Rodrigues, Cristiane Casagrande Denardin, Fabiane Moreira Farias, Daiana Silva Ávila

**Affiliations:** ^1^Laboratório de Pesquisa em Bioquímica e Toxicologia em Caenorhabditis elegans, Programa de Pós-Graduação em Bioquímica, Universidade Federal do Pampa, Uruguaiana, Brazil BR 472-Km 592-Caixa Postal 118, CEP 97500-970; ^2^Programa de Pós-Graduação em Ciências Farmacêuticas, Universidade Federal do Pampa, Uruguaiana, Brazil BR 472-Km 592-Caixa Postal 118, CEP 97500-970

## Abstract

Plants are widely used in folk medicine because of their pharmacological properties. *Ceiba speciosa*, popularly known as *paineira-rosa* or tree-of-wool, is a species found in the Northwest of Rio Grande do Sul, being native of the upper Uruguay River, Brazil. The tea obtained from the stem bark is employed in folk medicine to reduce cholesterol, triacylglycerides, and glucose levels. However, there are no studies in the literature proving its efficacy or the safety of its use. For this study, we used *Caenorhabditis elegans* as an animal model considering its advantages for risk assessment and pharmacological screenings. For the toxicological tests, *C. elegans* N2 (wild type) was treated with the aqueous extract of the stem bark of *C. speciosa* (ECE) at the first larval stage (L1) at concentrations of 5, 25, 50, and 250 *μ*g/mL. To evaluate biological activities, we challenged the extract for oxidative stress resistance in the presence of paraquat (0.5 mM), H_2_O_2_ (1 mM), and against glucose-induced toxicity. Our results demonstrated that ECE did not alter survival rate, pharyngeal pumping, and reproduction of the nematodes. The extract was not able to protect the nematodes against the toxicity induced by prooxidants. Notably, ECE protected against glucotoxicity by increasing worms' life span and by reducing glucose levels. On the other hand, ECE treatment did not reduce lipid accumulation induced by exogenous glucose feeding, as observed in worms which lipid droplets were tagged with GFP. Based on our results, we believe that the extract is indeed promising for further studies focusing on carbohydrates metabolism; however, it needs to be carefully evaluated since the extract does not seem to modulate lipid accumulation.

## 1. Introduction

Glucose toxicity refers to the biological effects of its excessive levels in cells and tissues due to metabolic alterations and production of advanced glycation end products (AGEs) [1]. AGES interact with lipids, proteins, and other molecules causing cell damage by inducing oxidative stress and cell death [2]. With the growing interest in finding natural antioxidants and drugs with beneficial action on glucose metabolism, many extracts from different plants and their different parts have been tested [3, 4]. However, the indiscriminate use of natural products has led to cases of intoxication [5]. Therefore, a safety assessment of natural products should be performed previously to medicinal use [4].

The scientific literature describes some medicinal properties for several species of the genus *Ceiba*. A study conducted by Leal et al. [6] reported a considerable variety of secondary compounds in the bark extract and antimicrobial activity against strains of *Staphylococcus aureus* which may be related to the presence of phytochemicals such as tannins and phenols. In addition, *Ceiba pentandra* (L.) Gaertn, commonly known as silk cotton tree, has been used by practitioners of traditional medicine in Northern and Eastern Nigeria for diabetes control.

In this same pharmacological context, *Ceiba speciosa* (A. St.-Hil.) has been used by the southern Brazilian population [7]. The species is found in the Northwestern Rio Grande do Sul, Brazil, being native in the Upper Uruguay vegetation [8]. Regarding its use in folk medicine, there are records for the use of barks and flowers for the treatment of heart disease, hypertension, and diabetes [9]. With the increasing interest in the bioprospection of natural products, especially of vegetal material originating from the Pampa Biome, the validation and application of alternative models for the evaluation of these natural products becomes relevant.

Among the animal models that have been used to investigate the toxicology and pharmacology of medicinal plants is the nematode *Caenorhabditis elegans*. This worm has a number of characteristics that make it not only relevant but also quite powerful as a model for this type of research [10]. *C. elegans* is easy and cheap to maintain in the laboratory with an *Escherichia coli* diet, has a short reproductive cycle (3 days) and a large number of descendants (~200), which allow the large-scale production of animals within a short period of time. The transparent body allows a clear observation of all cells in these animals. Notably, *C. elegans* shows a strong conservation in molecular and cellular pathways with respect to mammals and humans [11], including those signaling related to glucose and lipid metabolism [12, 13].

Notably, this nematode does not have specialized storage tissues such as adipocytes, and the energetic excess is stored in lipid droplets in enterocytes, hypodermis, and gonads [14, 15]. On the other hand, the absence of adipocytes removes central components in the control of energetic metabolism in mammals, which may be advantageous by facilitating the interpretation of other conserved factors and mechanisms involved in energy homeostasis [16, 17]. Thus, this study is aimed at evaluating the safety of the aqueous bark extract from *Ceiba speciosa* (ECE) as well as at verifying the glucose-reducing levels action attributed to this plant using *C. elegans* as an animal model.

## 2. Materials and Methods

### 2.1. Extract Preparation

The stem bark of *C. speciosa* was collected in the municipality of Santo Antônio das Missões, Rio Grande do Sul, Brazil, on April 2012. The plant material was identified by a botanist, and an *exsiccata* or botanical voucher was deposited in the Zoobotanic Herbarium Augusto Ruschi at the University of Passo Fundo, under the code RSPF 12637. *C. speciosa* barks were ground, and the aqueous extract was prepared by decoction in distilled water according to Malheiros et al. [18]. Since we have used the exact same batch of lyophilized extract, the phytochemical composition has been already reported [18]. To expose worms, the lyophilized material was diluted in water to obtain a stock solution at 10 mg/mL that was subsequently used for further dilutions.

### 2.2. *Caenorhabditis elegans* Maintenance

The *C. elegans* strains used in this study were N2 (wild type) and VS29 (hjSi56 [*vha-6p*::3xFLAG::TEV::GFP::*dgat-2*::*let-858* 3′UTR]) [19] which were obtained from *Caenorhabditis* Genetics Center (CGC) and maintained in NGM (nematode growth medium) seeded with *E. coli* OP50 at 20°C [20]. First larval stage worms were obtained by a synchronization process, which consists of exposing pregnant worms to the lysis solution (0.45 N NaOH, 2% HOCl p/v) to separate the eggs from the worms. After 14 h, the eggs hatched and released the L1 larvae, used for exposures. All experiments were performed at 22°C in a controlled humidified environment.

### 2.3. Treatment Protocol and LC_50_ Determination

A total of 2,000 L1 nematodes were exposed to different concentrations of *C. speciosa* bark extract (ECE-5 *μ*g/mL, 25 *μ*g/mL, 50 *μ*g/mL, and 250 *μ*g/mL) for 30 min at 22°C, in a homogenizer. After treatment, the nematodes were washed with 85 mM NaCl solution for 3 times in order to terminate the exposure, and then, nematodes were plated on NGM seeded with *E. coli* OP50. Twenty-four hours after exposure, the number of surviving nematodes was counted, and a survival curve was drawn [21]. The experiments were performed in duplicates. The results were expressed as % of control.

### 2.4. Brood Size

The nematodes were treated as described above and were maintained on NGM/*E. coli* OP50 plates until they reached larval stage L4. To evaluate progeny size, one nematode from each ECE treatment was transferred to a new plate containing NGM medium, and the total number of progeny was counted during the whole reproductive period [22]. The experiments were performed in triplicates and independently repeated three times. The results were expressed as % of control.

### 2.5. Pharyngeal Pumping

This assay is used to verify the dietary intake of the nematodes. 24 h after the treatment described previously, 5 nematodes from each experiment were transferred to new plates without bacteria. The pharyngeal pumps were counted for 1 min at the stereomicroscope [23]. The experiments were repeated in three independent assays. The results were expressed as % of control.

### 2.6. Oxidative Stress-Resistance Assays

Nematodes were pretreated at the L1 stage as described previously. After the last wash, worms were posttreated with the prooxidants paraquat (0.5 mM) or hydrogen peroxide (1 mM) for 30 min. Subsequently, nematodes were washed four more times and then transferred to NGM/*E. coli* OP50 plates. The number of surviving nematodes on each plate 24 h postexposure was scored to determine stress resistance [10]. The experiments were performed in duplicates and repeated at least 3 times. The results were expressed as % of control.

### 2.7. Glucose Toxicity

In the pilot test, we have used 2 glucose concentrations (2 and 4%) to verify whether they would reduce worms' life span. Plates containing NGM medium were inoculated with *E. coli* OP50 which were UV-inactivated. Thereafter, glucose solution was added on top of the dried *E.coli*. Based on pilot results, we have chosen glucose 4% for the next assays. Worms were pretreated with different concentrations of the extract (ECE 5 *μ*g/mL or 250 *μ*g/mL) for 30 min, washed to remove the treatments, and then transferred to NGM/UV-inactivated *E.coli* plates containing glucose 4% or vehicle. Then, 20 live worms at the same developmental stage were collected at the late L4 stage and transferred daily to new plates NGM UV-inactivated *E.coli* + glucose 4% or vehicle in order to prevent contamination or progeny. Live animals were scored until all animals died. The experiments were performed in triplicates, and three independent experiments were conducted. The results were expressed as % of control.

### 2.8. Glucose Levels

The glucose content was quantified by glucose oxidase method. Worms (N2) were treated as described in Section 2.7 and kept in glucose 4% until they reached the L4 stage. After these 48 h, worms were washed off with M9 buffer from the plates and centrifuged at 2500 rpm for 2 min. Worms were washed until removing all existing bacteria. Then, worms were frozen and thawed 3 times, sonicated with lysis buffer, and centrifuged. The supernatant (50 *μ*L) was transferred to a 96-well plate, and glucose levels were measured using a Labtest glucose colorimetric assay kit (Labtest Diagnostica S.A., Minas Gerais, Brasil). The optical density of each of the incubated samples (10 min at 37°C) was measured at 505 nm. For normalization of the data, protein was measured using Bradford colorimetric method and expressed as mg glucose/*μ*g protein. The experiments were performed in duplicates and normalized as % of control.

### 2.9. Glucose-Induced Lipid Accumulation

N2 and VS29 nematodes were treated as described in Section 2.7, and worms were kept on NGM/UV-inactivated *E.coli* OP50 plates containing or not glucose 4% until reaching the larval stage L4. After these 48 hours of glucose exposure, worms were transferred to slides containing levamisole, and images were acquired in a fluorescent microscope to observe the effect of glucose exposure on lipid accumulation. VS29 has a transgene which DGAT2 (acyl CoA:diacylglycerol O-acyltransferase 2) is fused to GFP. This enzyme transfers acyl groups and is localized in the lipid particles; then, lipid droplets can be observed. N2 was used as control to verify lipofuscin or autofluorescence production caused by glucose treatment.

### 2.10. Statistical Analysis

Statistical analysis was carried out by one-way analysis of variance (ANOVA), followed by post hoc Tukey test when the overall *p* < 0.05. For life span assay, a repeated measure two-way analysis of variance (ANOVA) followed by post hoc Tukey test was applied.

## 3. Results

### 3.1. Toxicity Evaluation of *C. speciosa* Aqueous Bark Extract

**Figure 1:**
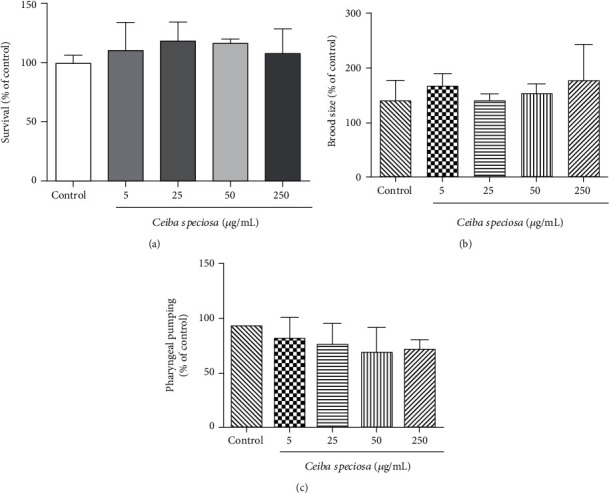
Toxicological evaluation of different concentrations of *C. speciosa* aqueous extract in (a) nematodes survival, (b) brood size, and (c) pharyngeal pumping. Values are expressed as mean ± S.E.M. of four experiments. Data were analyzed by using one-way ANOVA following by the Tukey test.

Figure 1(a) shows that acute exposure to different concentrations of ECE did not alter *C. elegans* survival rate (up to 250 *μ*g/mL), thus suggesting that no toxic effects occurred under the experimental conditions tested (acute). Based on that, we decided to use the concentrations of 5 *μ*g/mL, 25 *μ*g/mL, 50 *μ*g/mL, and 250 *μ*g/mL for further assays. In addition, we did not find any toxic effect of *C. speciosa* bark extract on worms' reproduction, as brood size did not change (Figure 1(b)). In order to verify whether ECE would affect food ingestion *per se*, we analyzed pharyngeal pumping, which was not changed as well (Figure 1(c)).

### 3.2. Stress Resistance

**Figure 2:**
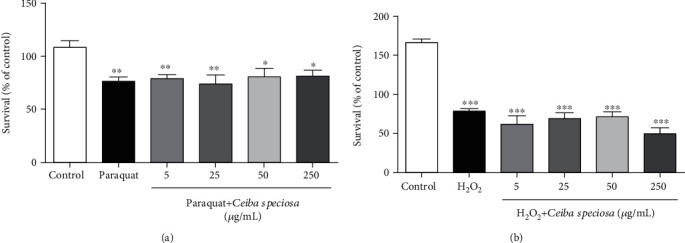
Effect of different concentrations of ECE treatment in oxidative stress resistance. (a) Response to oxidative stress in paraquat posttreated nematodes (0.5 mM) and pretreated with ECE. Values are expressed as mean ± S.E.M. of six experiments. (b) Response to oxidative stress in post-H_2_O_2_ treated nematodes (1 mM) and pretreated with the ECE. Values are expressed as mean ± S.E.M. of three experiments. Data were analyzed by using one-way ANOVA following by the Tukey post hoc test. ^∗^ indicates *p* < 0.05; ^∗∗^ indicates *p* < 0.01; ^∗∗∗^ indicates *p* < 0.001 when compared to the control group.

To verify a possible stress resistance conferred by ECE, we exposed worms to different prooxidants: paraquat (a pesticide, Figure 2(a)) or hydrogen peroxide (H_2_O_2_, Figure 2(b)). Our results demonstrate that ECE did not protect against the damage induced by these prooxidants at any of the tested concentrations.

### 3.3. ECE Treatment Protected against Glucose Toxicity

**Figure 3:**
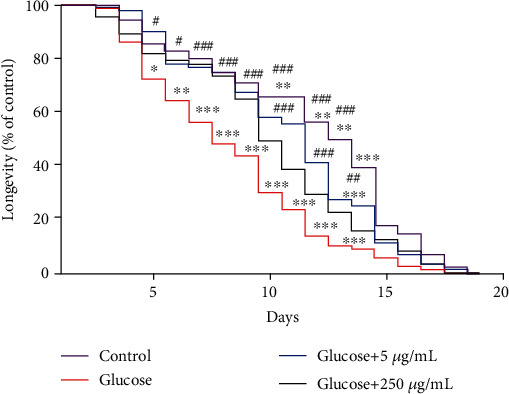
Effect of different concentration of ECE on glucose-induced toxicity in worms' life span. Values are expressed as mean ± S.E.M. of four experiments. Data were analyzed by using a repeated measures two-way ANOVA followed by the Tukey posttest. ^∗^ indicates *p* < 0.05; ^∗∗^ indicates *p* < 0.01; ^∗∗∗^ indicates *p* < 0.001 when compared to the control group. ^#^ indicates *p* < 0.05; ^##^ indicates *p* < 0.01; *^###^* indicates *p* < 0.001 when compared to the glucose group.

**Figure 4:**
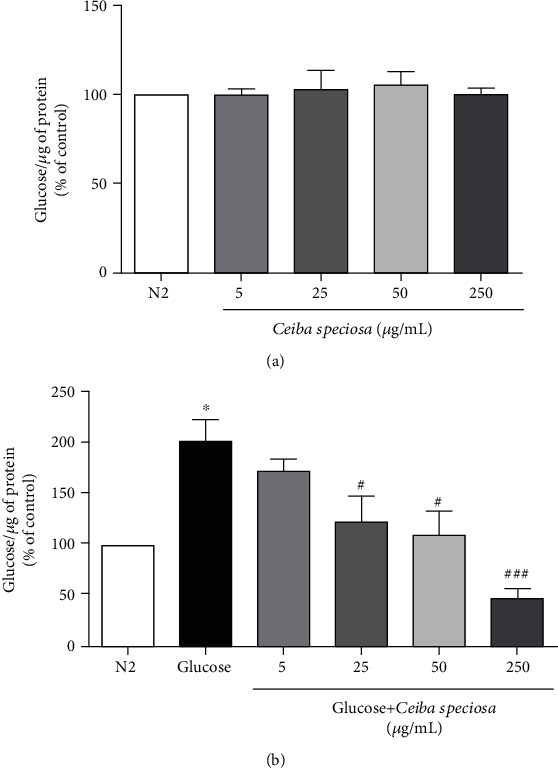
Glucose levels in (a) following ECE treatment per se and (b) worms treated with ECE and exposed to glucose 4%. Values are expressed as mean ± S.E.M. of four experiments. Data were analyzed by using one-way ANOVA following by the Tukey post hoc test. ^∗^ indicates *p* < 0.05 when compared to the control group. ^#^ indicates *p* < 0.05; *^###^* indicates *p* < 0.001 when compared to the glucose group.

In order to verify the protection of ECE treatment against glucose toxicity, we analyzed life span. Post hoc analysis of the data depicted in Figure 3 has revealed that 4% glucose induced a reduction in worms' life span from day 5 until day 14. The treatment with ECE at both concentrations (5 and 250 *μ*g/mL) was effective against the glucose-induced reduction in life span starting on day 6 until day 12. Two-way ANOVA demonstrated a significant interaction between treatment × days (*F*_(54,162)_ = 4.418; *p* < 0.001), thus indicating a protection conferred by ECE throughout the life span. In addition, glucose 4% administration significantly increased glucose levels, and the treatment with ECE was effective by reducing this elevation (Figure 4(b)). Notably, ECE per se treatment did not alter glucose levels (Figure 4(a)).

**Figure 5:**
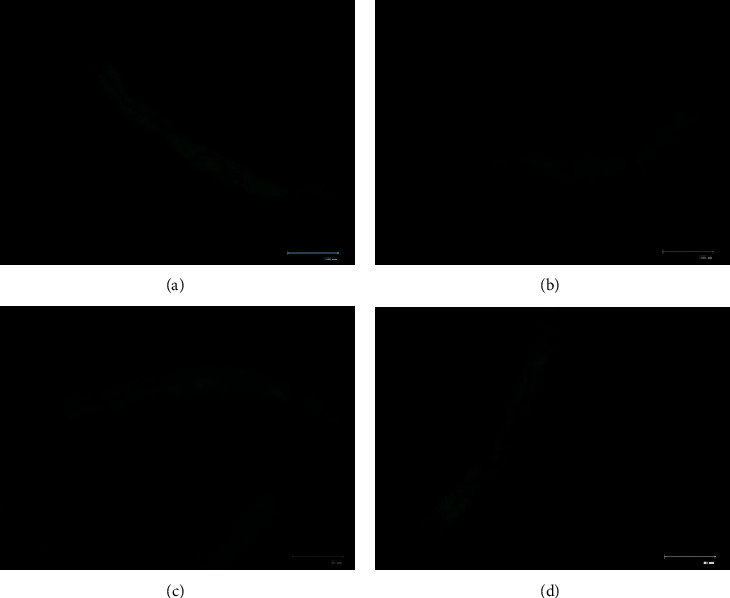
Representative images of N2 worms following glucose-induced lipid-accumulation and ECE treatment to observe autofluorescence: (a) untreated worms; (b) worms treated with glucose 4%; (c) worms treated with ECE 5 *μ*g/mL+ 4% glucose; (d) worms treated with ECE 250 *μ*g/mL+ 4% glucose.

**Figure 6:**
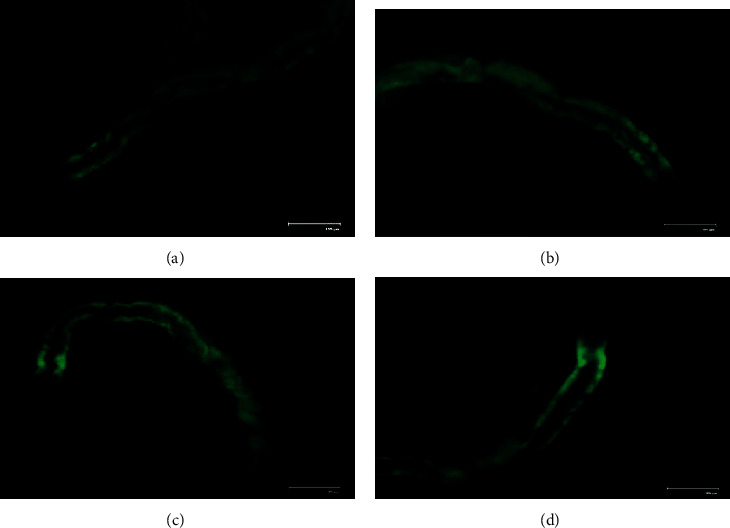
Representative images of VS29 worms following glucose-induced lipid-accumulation and ECE treated worms: (a) untreated worms; (b) worms exposed to glucose 4%; (c) worms treated with ECE 5 *μ*g/mL+ 4% glucose; (d) worms treated with ECE 250 *μ*g/mL+ 4% glucose.

Furthermore, we took advantage of a GFP-tagged strain and observed lipid accumulation induced by glucose vs. ECE pretreatment. In wild-type (N2) worms, no increase or decrease in fluorescence intensity caused by lipofuscin production was evidenced, even in those worms exposed to high glucose levels (Figures 5(a)–5(d)). On the other hand, in VS29 mutants, a stronger fluorescence around the gut following glucose 4% exposure was detected, therefore indicating an increase in lipid droplets accumulation (Figure 6(b)). However, ECE treatment did not reduce lipid accumulation at any of the concentrations tested since no significant reduction in GFP fluorescence was evidenced (Figures 6(c) and 6(d)).

## 4. Discussion

In the present work, the safety and pharmacological activity of *C. speciosa* aqueous bark extract were studied, since little scientific knowledge about its effects are described in the literature. The consumption of this plant occurs in the Northwestern region of the State of Rio Grande do Sul, Brazil, which consumes it as a tea due to its alleged beneficial effects in humans [9]. Our work verified that ECE is safe and protected against glucose-induced toxicity by reducing its levels and the associated consequences in *C. elegans* life span.

The antioxidant potential of the extract has been previously demonstrated *in vitro* through the DPPH radical scavenging capacity and demonstrated a good scavenging potential [18, 24]. The phytochemical analysis of the extract used in this study was published by Malheiros and cols and revealed that flavonoids (quercetin, rutin, and kaempferol) and phenolic acids (gallic, chlorogenic, ellagic, and caffeic) are present [18, 25].

*In vivo*, however, the extract did not show the same antioxidant performance. We challenged ECE against two powerful prooxidants, paraquat and H_2_O_2_, which have significantly increased nematodes mortality. Paraquat is an herbicide widely used in agriculture, but its biochemical mechanism responsible for its toxicity is not fully understood. It is known that paraquat is a superoxide radical generator (O2·), which can produce other reactive species such as hydrogen peroxide (H_2_O_2_) and hydroxyl radical (·OH), which are unstable and react quickly with fatty acids, causing lesions in the membranes, proteins, and DNA. H_2_O_2_ is one of the most versatile oxidants in existence, superior to chlorine, chlorine dioxide, and potassium permanganate; through catalysis, H_2_O_2_ can be converted to hydroxyl radical (·OH). It can easily pass through cell membranes [26, 27]. Our results demonstrated that despite the antioxidant action *in vitro*, the treatment with ECE was not able to reduce the toxicity induced by these prooxidants. *In vivo*, molecules can be transformed, sometimes losing activities presented in some *in vitro* assays, particularly those without tissue.

*C. elegans* contains many key metabolic-related components such as oxidative stress and insulin signaling pathways, making it a useful platform to improve our understanding of complex diseases such as metabolic syndrome [13]. Glucose is the main energy source and key regulator of animal metabolism; however, a chronic abundance of glucose will have deleterious effects on cellular and tissue functions [28]. Carbohydrate excess activates several lipogenic enzymes contributing to deregulation in lipid metabolism and insulin resistance development [29]. Furthermore, high levels of glucose induce cell injury of mammalian hepatocytes and pancreatic cells through molecular mechanisms of endoplasmic reticulum stress, oxidative stress, and mitochondrial impairment [29, 30]. Moreover, chronic hyperglycemia can deteriorate cognitive function and impair memory [31].

*C. elegans* is a suitable model organism to study glucose toxicity, once high glucose conditions impair their life span by increasing ROS formation and AGEs modification of mitochondrial proteins in a DAF-2 independent manner [32]. Furthermore, glucose-enriched diet significantly decreases worms' life span by inhibiting the translocation of DAF-16 and HSF-1 transcription factors that are involved in antioxidant response [33]. On the other hand, glucose restriction extends *C. elegans* life span by increasing the expression of proaging and antioxidant genes [34].

We have found promising results when we challenged ECE-treated worms against high glucose levels. The extract was able to protect from the reduced longevity and to diminish whole body glucose levels induced by high glucose exposure. A similar effect has been observed in the alloxan-induced diabetic rats treated with *B. ceiba* bark methanolic extract [35]. Furthermore, the alcoholic extract of *C. pentandra*, a species taxonomically related to *C. speciosa* which is commonly used in North Africa, has been used to control hyperglycemia in diabetic patients [36]. Notably, its alcoholic extract has been tested in rats, and it has been proven that besides safe, decreases plasma glucose levels following oral administration [37].

In relation to mechanism of action, the treatment with *C. pentandra* decreased glucose levels in diabetic rats through enhanced glucose utilization and regulation in insulin levels [38, 39]. Similar to *C. pentandra*, we believe that the treatment with ECE could enhance glucose utilization and stimulate another form of energy storage, like glycogen or trehalose. Notably, *C. elegans* can synthesize saturated and unsaturated fatty acids and is capable of storing energy as lipids (triglycerides, phospholipids, sphingolipids, etc.). Up to 35% of *C. elegans* dry body mass is lipids, being triacylglyceride fat deposits the major energy storage molecules, depending on diet and life stage [16]. To analyze that, we have used a transgenic strain which acyl CoA:diacylglycerol O-acyltransferase 2 is fused to GFP. This enzyme transfers the acyl groups and is localized in the lipid particles; then, lipid droplets can be observed *in vivo*. As expected, our results have shown that high glucose administration increased lipid accumulation in worms. However, ECE treatment failed to reduce this storage, therefore indicating that glucose levels reduced by the extract may have been converted to lipids.

Finally, we observed that acute exposure at different concentrations of ECE did not cause toxicological effects on worms, as analyzed by survival rate, brood size, and pharyngeal pumping. These findings confirm that worms were ingesting the extract and feeding normally, indicating that the use of these concentrations is safe, considering the experimental conditions tested. Previous *in vitro* data have also demonstrated that this same extract did not reduce leukocytes viability and did not cause genotoxicity [18]. *C. speciosa* is a plant widely used by southern Brazilians; therefore, the safety of its use by this population is germane. Thus, for the first time, it was demonstrated that the aqueous bark extract of *C. speciosa* has low toxicity and has potential against glucose toxicity, thus suggesting that ECE may be useful against hyperglycemia in mammals. Therefore, additional studies will be carried out by our group with the purpose of expanding the hypoglycemic effects of *C. speciosa* and its mechanisms.

## Data Availability

All authors declare the availability of the use of data from this manuscript by the journal Antioxidants and Prooxidants: Effects on Health and Aging 2020.

## Supplementary Materials

Supplementary Materials The supplementary material includes the graphical abstract of the manuscript.
