# Supplementary figures and images for: Aqueous Bark Extract of Ceiba speciosa (A. St.-Hill) Ravenna Protects against Glucose Toxicity in Caenorhabditis elegans
Source: Oxid Med Cell Longev. 2020 Oct 8;2020:1321354. doi: 10.1155/2020/1321354 (PMC7568133; doi:10.1155/2020/1321354)

Graphical abstract

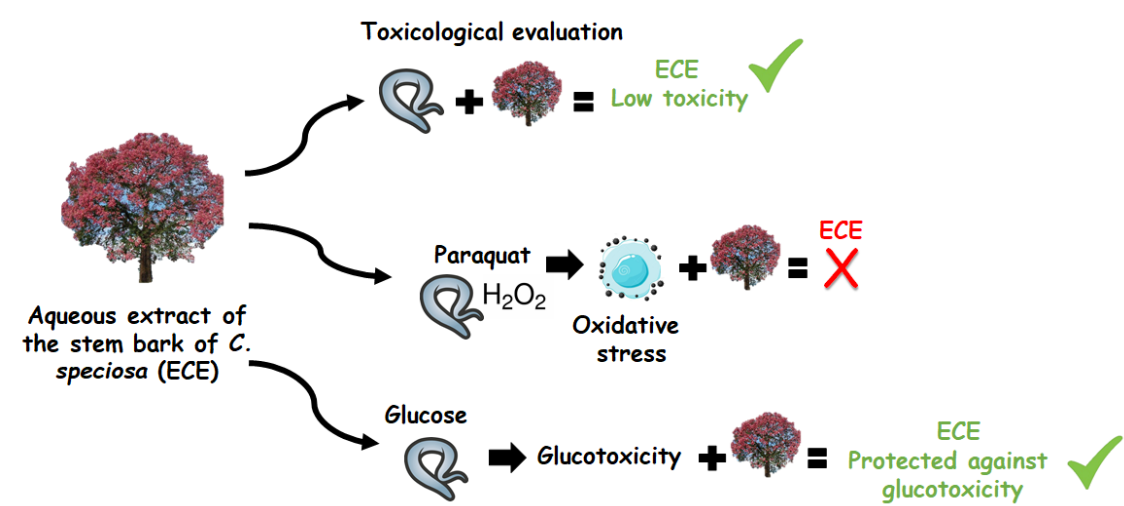

Supplement: Supplementary Materials — The supplementary material includes the graphical abstract of the manuscript. [file 1321354.f1.pdf]
